# On the Recent Advances in the Theory of Vision

**Published:** 1872-04

**Authors:** H. Helmholtz

**Affiliations:** The Royal Society of London


ART. V.—*On tho recent Advances in the Theory of Vision.* By H. HELMHOLTZ, (*of the Royal Society of London.*)

Translated from *Les Annales d' Oculistique*, BY F. W. ABBOTT, M. D.

(*Continued.*)

Physics demonstrates that light is an oscillatory motion of an elastic medium, called the *luminiferous ether*, extending throughout space, whose vibrations are diffused in the form of waves; it is a motion analogous to that of the circular waves which are formed upon the surface of still water into which a stone has just fallen; we may also compare this motion to the disturbance which sound makes in diffusing itself in our atmosphere. But the propogation of light and the oscillations of the particles set in motion by the luminous waves take place with a velocity incomparably superior to those of the water and the air in the examples which I have just cited.

The systems of luminous waves which emanate from the sun differ considerably among themselves in their dimensions, just as upon the water the waves form sometimes little ripples a few inches long, sometimes immense billows whose summits embrace abysses from sixty to a hundred feet in width. Now, just as the waves of a liquid surface, whether they are high or low, short or long, do not differ among themselves in nature, but only in size, so also, the systems of luminous waves which emanate from the sun differ in force, and length of wave, but all execute analogous motions. All present the same remarkable physical properties, reflection, refraction, interference, diffraction, and polarization, and this analogy, which subsists in spite of certain differences attributable to the difference of wave-lengths, enables us to affirm that in all these systems of waves, the vibratory motion of the luminiferous ether is of the same kind. We remark particularly that the phenomena of interference, where the light reinforces or destroys itself when we join to it light of the same kind which has run a longer or a shorter course, prove that all these luminous emissions consist in an oscillating undulatory motion; we remark also the phenomena of polarization which, varying with the side whence they are observed, enable us to affirm that the oscillations of the particles of ether are perpendicular to the direction in which the ray is propagated.

All the different rays of which we have just spoken have this in common; to warm the terrestial bodies which they strike, and to produce a sensation of heat upon our skin.

But our eye, on the contrary, perceives as light only a part of these vibrations of the ether. It is not effected by the systems which present great lengths of wave, and which we may compare to the long billows of the ocean; so they receive the designation of *invisible calorific rays.** These are rays of the kind which a stove emits when it warms us without being heated to redness.

On the other hand our eye perceives so feebly the rays of short wave-length, comparable to the ripples produced by a light wind upon the surface of a pond, that these rays also are generally considered invisible, and so they have been called *invisible chemical rays.*

Between the waves of the ether which are too short and those which are too long, there are some of a mean length which strongly effect our eye without differing essentially from the invisible heat rays and the invisible chemical rays. They are only distinguished by the different value of their wave-lengths, and by the physical relations resulting therefrom. We give them the name of *light,* for the reason that they alone affect our retina.

When we take into consideration the calorific quality of these rays, we designate them also under the name of *luminous heat,* and as they produce upon our skin a sensation entirely different from that which they produce in the eye, heat was considered, until thirty years ago, as proceeding from a radiation of an entirely different nature from light. But, as has been demonstrated by researches made since with the greatest care by physicists, light and heat are identical, and it is impossible to separate them in the luminous rays of the sun. Whatever may be the optical operations which they are made to undergo, it is impossible to diminish the luminous power of the rays without lessening at the same time and in the same degree their calorific and chemical effects. Every operation which suppresses the oscillatory motions of the ether sup-

* [Though the term is by no means faultless, those rays both ultra-red and ultra violet, which are incompetent to excite vision are called *invisible rays.* In strictness we cannot speak of rays being either visible or invisible; it is not the rays themselves, but the objects which they illuminate which become visible. Tyndall. Notes on light. No. 249.]

presses naturally, also, all the effects of these motions, that is to say, light, heat, chemical action, the excitation of flourescence, etc.

Those vibrations of the ether which strongly affect our eyes, and which we call light, excite in us the impression of different colors, according to the difference of their wave-lengths. The vibrations which present a great wave-length appear to us as *red*, then come successively the colors *orange*, *yellow*, *green*, *blue*, *indigo* and *violet*, this last color corresponding to the least wave-length capable of producing a luminous impression. This succession of colors is well known: it constitutes the rain-bow; we see it likewise when we look at a light through a glass prism, and it is the same series of colors which forms the colored fires of the diamond. In fact, in transparent bodies light is analyzed owing to the difference in the wave-lengths of the different colors, which produces a difference of refraction, whose effect is to isolate these colors one from another. These simple colors, which we obtain in the greatest purity in projecting, by the aid of a glass prism, the spectrum of a line of solar light, are at the same time the most brilliant and the most saturated that are to be seen.

The mixture of several of these simple colors give the impression of a new color, generally more or less whitened. We obtain the impression of white when all are mixed exactly according to the proportion which they present in solar light. In the mixtures, according as the rays which predominate are of great, medium, or small wave-length, the color obtained is of a reddish, greenish, or bluish white. In suffices to have seen a painter work to know that the mixture of two colors gives a third. In spite of the numerous differences of detail between the results of the mixture of colored rays and those of the mixture of the colors for the painter, the phenomena occur upon the whole, in an analogous manner in both cases. Whether it be upon a part of our retina, or upon a white screen, that we superimpose rays of two different colors at once, in one case as in the other, in the place of two colors we see only one, and this *resulting color* differs always more or less from the two colors which enter into its composition.

The most striking difference between the mixture of colors for the painter and the mixture of colored rays, consists in this, that painters obtain green by the mixture of blue and yellow, while the mixture of yellow and blue light gives white.

So, in general, different mixtures awaken in us the sensation of different colors. But the number of perceptible colored differences is much less than that of the different mixtures of luminous rays which the external world may send to our eye. For the retina, the mixtures of scarlet and greenish blue rays, greenish yellow and violet, yellow and ultra-marine blue, red, green and violet, or finally of all the colors of the spectrum; all produce the same sensation of white. Physically, these mixtures are, neverthe-less, very different; and *we are not even able to demonstrate any kind of physical resemblance between these different mixtures* if it is not the identity of their action upon the eye. Thus, for example, a surface illuminated with red and with greenish blue will become black in a photograph, while another illuminated with yellowish green and violet will become very light [*claire*] although both surfaces are alike white to the eye. Moreover, if we successively illuminate colored bodies by the aid of these different white lights, their coloring will appear different each time. Finally it suffices to look at these different lights through a prism or a colored glass to recognize them as different.

As is the case with white light so we may, by using very different mixtures of colored light, reproduce other colors, especially when they are not extremely saturated; the resulting colors thus obtained may be identical to the eye, whatever may be the difference of their composition, and, consequently, that of their physical or chemical properties.

We are indebted to Newton for a process which enables to represent in a very simple manner, the system of colors of which the eye can appreciate the difference, and by the aid of which the law of the mixture of colors is expressed in a relatively simple manner. Let us imagine the colors of the spectrum suitably arranged around the circumference of a circle, commencing with the red and following the series of the colors of the rainbow by insensible shadings to the violet; we unite the red and the violet by a purple, passing on the one hand into the blueish violet and on the other into the yellowish scarlet of the spectrum. We put white in the centre of the circle, and upon the radii which go from the centre to the periphery, we dispose by insensible gradations the colors which may be produced by the mixture of white with the saturated peripheral colors. The chromatic circle thus constructed represents all the variations which the colors can present, the intensity moreover remaining equal.

Now—this can be demonstrated—if we properly arrange the colors upon such a table, and if we properly choose the measure of their intensities, it suffices to construct the centre of gravity to find the position of the color which results from the mixture of two colors of the table. The construction is made by replacing the weights by the luminous intensities. Thus, upon the chromatic table properly constructed, all the resulting colors which two colors can make are situated upon the straight line which unites these two colors, and these resulting colors are by so much nearer one of these composing colors as they contain more of this one and less of the other.

I remark that, in such a disposition, the colors of the spectrum, which, being the most saturated which exist, ought to be placed the farthest possible from the white, that is to say, upon the circumference of the table, do not adjust themselves exactly in a circle. The circumference of the figure presents three projections, in the red, the green, and the blue, so that the whole very nearly resembles a triangle with rounded corners. While the red, the green, and the blue occupy the angles, the sides of the triangle contain the transitions from the red to the green through the yellow, from the green to the indigo through the greenish blue, and from the indigo to the red through the violet and purple.

With his representation of the system of colors, analogous to that which we have just described, Newton had no other object than to give a comprehensive idea of the complicated facts relative to our subject. Very recently, Maxwell has been able to demonstrate, even from a quantitative point of view, the accuracy of the propositions comprised in this representation. He accomplished this by means of discs whose different sectors bear different colors, the mingling of which is effected by means of a rapid rotation. When one of these discs turns so swiftly that the eye can no longer follow the different sectors, the colors of these sectors blend into one uniform resulting color, and the quantity of the light relative to each one of the colors may be measured directly by the size of the sector which it covers. Now we may demonstrate experimentally that the resulting colors thus obtained are identical with those which the simultaneous illumination of the same surface with the same colors would give. It is thus that measure has been able to penetrate into the study of colors, which appeared inaccessible to it, and the qualitative differences of colors have been brought into the domain of quantitative circumstances.

We see that all the differences of colors become reduced to three; those of *tone*, *saturation*, and *intensity*. The differences of *tone* are those which exist between the different colors of the spectrum, and which we designate by the names of red, yellow, green, blue, violet and purple, so, relatively to tone, the colors form a continuous series, such as we obtain in uniting by purple the extreme colors of the rainbow, and such as we have represented upon the circumference of the chromatic table. *Saturation* presents its maximum in the simple colors of the spectrum at least in so much as the sensations produced solely by external light are in question; we shall see farther on that in sensation saturation may attain a still higher degree. Every addition of white to the spectrum colors diminishes their saturation. It is thus that rose is identical with whitened purple, flesh color with whitened scarlet; the pale green, pale yellow, pale blue, etc., are the same colors little saturated, or mingled with white. All the composite colors are, as a general rule, less saturated than the simple colors of the spectrum. Finally, the differences of *brightness* or *intensity* have not been represented upon the chromatic table. Only so long as we consider colored light, do these differences of intensity present a quantitative character. Black is then only darkness or simply the absence of light. It is otherwise when we look at colored objects; black then corresponds as well as white to a manner of reflecting light, and for this reason, in ordinary language, it is considered a color as well as white. There also exists a series of denominations for the colors of feeble intensity. We call them dark or deep, or sombre [foucées] when they are saturated, and *grayish* [*grises*] when they are whitened. Thus the *dark blue* is a blue of feeble intensity and saturated; *grayish blue* is a blue of feeble intensity and whitened. For certain colors the employment of special names affords greater brevity.

Thus reddish brown, brown, olive green are feeble gradations, more or less saturated of red, yellow and green.

We have just seen how, in relation to sensation, the objective differences in the composition of light may all be reduced to differences of *tone, saturation* and *intensity*, and how language conforms itself to this distinction. We may express this triple difference in still another manner.

I have said before that the chromatic table properly constructed resembles a triangle, let us suppose for an instant that it forms a rectilinear triangle; we will consider later the error which this supposition involves. We agree with Maxwell that red, green, and ultramarine occupy its angles. It follows, from the law of the mixture given above, that all the colors of the interior and of the sides of the triangle may be obtained by the mixture of the three colors which occupy the angles. Thus *all the differences of the colors correspond to different mixtures of three fundamental colors.* It is best to take for fundamental colors those indicated by Maxwell. It would be a bad choice to take, with the ancient authors, yellow and blue which have been chosen as fundamental colors from the mixtures of colors for painting, in fact, the yellow and blue rays do not give green by their mixture.

The peculiarities of this method of reducing all the varieties of light to the mixtures of three fundamental colors becomes more clear by comparing it with what occurs in an analagous manner in the ear.

We know that sound, as well as light, is an oscillatory movement whose vibrations are diffused in the form of waves, in like manner as with light, we have to distinguish in sound systems of waves of different wave-lengths, which excite in our ear impressions of diverse qualities; the waves of great length produce low sounds, those of short length high sounds. Like the eye the ear may be struck at the same time by several systems of waves, that is to say, by several sounds. But in the ear these sounds do not mingle in the same way as colors, whose simultaneous sensations produce colors which we have called resulting. We cannot replace the simultaneous action of *do* and *mi* by another sound like *re*, without completely changing the impression; while the eye does not notice the substitution of orange in the place of a mixture of red and yellow. The most complicated harmony of an orchestra would produce in us a different sensation, should we replace one of the sounds which compose it with one or two others. For a cultivated ear, there exists no harmony which is just exactly like another composed of other sounds. If the ear behaved in relation to sounds as the eye in relation to colors, it would be possible to replace any harmony, whatever it might be, by the combination of three simple sounds, one very low, the second of medium pitch, and the third very high. It would be sufficient to vary the relative intensity of these three sounds and all music could be reduced to the combinations of three sounds.

We find, on the contrary, that a harmony remains invariable to the ear only so long as the intensity of each one of the sounds of which it is composed remains the same. To define a harmony with perfect accuracy it would be necessary, therefore, to determine exactly the intensity of all the sounds of which it is composed. So also, the physical nature of a kind of light can be completely determined only by measuring the intensity by all the simple colors which this light contains. Now, the light of the sun, of the greater part of the stars, and of flames contain an immense number of different colors which shade into each other by insensible gradations. To define such light exactly, from a physical point of view, it would be necessary, therefore, to measure the intensity of innumerable elements, while in sensation we only distinguish the variation in the intensity of the elements.

The skilled musician is able to hear immediately in a harmony the note produced by each instrument of a complete orchestra. The physicist cannot recognize immediately, by simple sight, the composition of a light, he is obliged to have recourse to a prism, which decomposes the light. But then the composition of the light becomes manifest, and we may recognize the light of the different fixed stars, from the bright and dark lines of their spectrum ; we recognize equally the chemical elements which are contained in terrestrial flames, the burning atmosphere of the sun, of the fixed stars, and the nebulæ. Spectrum analysis, the most brilliant discovery of modern times, which has rendered the most remote regions of the sky accessible to chemical analysis, rests solely upon the fact that the light from each luminous source contains certain ineffaceable physical peculiarities which it preserves even in mixtures.

It is very interesting to know that there are some eyes which reduce the differences of the colors to a still more simple system, that of the mixture of only two fundamental colors. We say that these eyes, which are very frequently encountered, are affected with Daltonism, or better still *color blindness,* because they confound colors which appear very different to ordinary eyes. These eyes are not less apt in distinguishing the other colors, perhaps even a little more accurately, than normal eyes. Generally these eyes are affected with *anerythropsy* (*no-red-seeing*), that is to say, in their system of colors it is the red that is lacking, and, consequently, all the variations which the addition of red gives to the other colors. All the differences of color appear differences of blue and of green, to which they give the name of yellow. Thus scarlet, bright red, pale and greenish blue appear the same to them, or at most present only differences in intensity. So also purple, violet, and blue appear the same to them, likewise red, orange, yellow and green. The scarlet flowers of the geranium appear to them of the same tone as its leaves; they cannot distinguish between the red and the green lanterns which are used for signals upon railroads; they do not see the red extremity of the spectrum; very saturated scarlet appears almost black to them, to such a degree that a Scotch priest, suffering from color blindness, by mistake had a cassock made of scarlet cloth.

We encounter, in the perception of colors, some curious inequalities in the expanse of the retina. Firstly, every one is blind to red near the limit of the field of vision. If we move a geranium flower to and fro near the border of the visual field, we see its motion, but we do not distinguish its color which is confounded with that of the foliage of the plant, for generally, in indirect vision red appears much darker.

In the second place, we have seen that around the fovea centralis the retina is of a yellow tint, it follows from this that blue appears a little darker in the centre of the field of vision. This is especially striking in mixtures of red and blueish green. When such a mixture appears white to direct vision, it appears blue in the peripheral parts of the visual field; inversely, when these mixtures appear red to direct vision they appear white as soon as they recede from the point of fixation.

By a process which I have noticed in a preceding chapter, these irregularities pass unpercieved owing to the constant motion of the eye. With regard to the pale and whitened colors which the external world ordinarily presents to us, we know to what impressions of direct vision the impressions received indirectly correspond, and we interpret the latter so as always to appreciate the colors in the way in which they are presented to direct vision. Unusual colored mixtures, or especial attention is necessary for the difference to become appreciable.

*To be Continued.*

